# Physical activity, sedentary behaviors, and estimated insulin sensitivity and secretion in pregnant and non-pregnant women

**DOI:** 10.1186/1471-2393-11-44

**Published:** 2011-06-16

**Authors:** Anna Gradmark, Jeremy Pomeroy, Frida Renström, Susanne Steiginga, Margareta Persson, Antony Wright, Les Bluck, Magnus Domellöf, Steven E Kahn, Ingrid Mogren, Paul W Franks

**Affiliations:** 1 Genetic Epidemiology and Clinical Research Group, Department of Public Health and Clinical Medicine, Section for Medicine, Umeå University Hospital, Umeå, Sweden; 2 NIDDK/NIH, Diabetes Epidemiology and Clinical Research Section, Phoenix, AZ, USA; 3 Department of Clinical Sciences, Genetic & Molecular Epidemiology Unit, Lund University, Malmö, Sweden; 4 Department of Nutrition, Harvard School of Public Health, Boston, MA, USA; 5 Free University Medical Center, Amsterdam, the Netherlands; 6 Department of Clinical Science and Department of Nursing, Umeå University Hospital, Umeå, Sweden; 7 MRC Human Nutrition Research, Cambridge, UK; 8 Department of Pediatrics, Umeå University Hospital, Umeå, Sweden; 9 Division of Metabolism, Endocrinology and Nutrition, Department of Medicine, VA Puget Sound Health Care System and University of Washington, Seattle, WA, USA; 10 Department of Clinical Sciences, Obstetrics & Gynecology, Umeå University Hospital, Umeå, Sweden

**Keywords:** pregnancy, physical activity, sedentary time, β-cell response, insulin sensitivity

## Abstract

**Background:**

Overweight and obesity during pregnancy raise the risk of gestational diabetes and birth complications. Lifestyle factors like physical activity may decrease these risks through beneficial effects on glucose homeostasis. Here we examined physical activity patterns and their relationships with measures of glucose homeostasis in late pregnancy compared to non-pregnant women.

**Methods:**

Normal weight and overweight women without diabetes (N = 108; aged 25-35 years) were studied; 35 were pregnant (in gestational weeks 28-32) and 73 were non-pregnant.

Insulin sensitivity and β-cell response were estimated from an oral glucose tolerance test. Physical activity was measured during 10-days of free-living using a combined heart rate sensor and accelerometer. Total (TEE), resting (REE), and physical activity (PAEE) energy expenditure were measured using doubly-labeled water and expired gas indirect calorimetry.

**Results:**

Total activity was associated with reduced first-phase insulin response in both pregnant (Regression r^2 ^= 0.11; Spearman r = -0.47; p = 0.007) and non-pregnant women (Regression r^2 ^= 0.11 Spearman; r = -0.36; p = 0.002). Relative to non-pregnant women, pregnant women were estimated to have secreted 67% more insulin and had 10% lower fasting glucose than non-pregnant women. Pregnant women spent 13% more time sedentary, 71% less time in moderate-to-vigorous intensity activity, had 44% lower objectively measured total activity, and 12% lower PAEE than non-pregnant women. Correlations did not differ significantly for any comparison between physical activity subcomponents and measures of insulin sensitivity or secretion.

**Conclusions:**

Our findings suggest that physical activity conveys similar benefits on glucose homeostasis in pregnant and non-pregnant women, despite differences in subcomponents of physical activity.

## Background

During late pregnancy insulin sensitivity declines by 40-60% compared to pre-gestational insulin sensitivity [1] and insulin secretion typically increases, which is a compensatory response and helps maintain glucose homeostasis [1]. The mechanisms driving insulin resistance in a healthy pregnancy, as well as in a pregnancy complicated by hyperglycemia, are complex and differ from the non-pregnant scenario [2]. In addition to a decline in insulin sensitivity, other features of pregnancy such as changes in diet or physical activity can impair systemic glucose regulation. One common barrier to physical activity in pregnant women is pregnancy-related pain; the prevalence of pelvic girdle pain or low back pain in pregnancy has been reported to be as high as 45% [3].

Physical activity is a complex exposure with multiple subcomponents. Some of the subcomponents measured with objective methods such as accelerometers have been shown to be associated with glucose regulation in non-pregnant populations. Total physical activity as assessed by accelerometry is positively associated with insulin sensitivity in non-pregnant adults [4]. All studies to date examining associations between physical activity and glucose regulation during pregnancy have relied on subjective self-report measures of physical activity. Self-reported vigorous activity prior to pregnancy and light-to-moderate activity during pregnancy are associated with a reduced risk of hyperglycemia during pregnancy [5].

Regular physical activity is known to improve peripheral insulin sensitivity [6] and, in non-pregnant adults, increasing insulin sensitivity through lifestyle modification improves glucose tolerance and may aid in preserving β-cell function [7]. However, it is unclear if the effects of physical activity on glucose homeostasis observed outside pregnancy are the same as during pregnancy. Although lifestyle modification such as diet and physical activity to limit excessive weight gain is the front-line therapy for GDM [8], it is unknown if lifestyle modification can prevent GDM. Several intervention studies have been performed in pregnancy [9-12]. Whilst some of these studies sought to quantify the amount and intensity of exercise performed during the hours of the intervention, none to date has adequately assessed overall physical activity energy expenditure (PAEE) or non-exercise activity. This point is important, as people undergoing exercise interventions may compensate by reducing physical activity during other parts of the day [13]. Thus, there is a requirement for studies that define the appropriate dose, intensity, and mode of physical activity during pregnancy to maintain optimal weight gain and glucose regulation, so that guidelines to help prevent GDM are evidence-based.

A goal of this study was to explore the relationships between subcomponents of physical activity (e.g., PAEE, total activity, time spent in moderate-to-vigorous intensity activity, and sedentary time) and estimates of insulin sensitivity and β-cell responses in pregnant and non-pregnant women. Most importantly, we sought to determine if the relationships between subcomponents of physical activity and estimates of insulin sensitivity and β-cell responses differ in pregnant versus non-pregnant women.

## Methods

### Participants

A total of 108 women were studied at the baseline visit, of whom 35 were pregnant and 73 were not pregnant. Non-pregnancy was confirmed by a urine pregnancy test upon arrival at the Clinical Research Center at Umeå University Hospital. Pregnant and non-pregnant women were broadly matched on demographic characteristics such as age, weight and place of residency. All women lived in eastern Västerbotten, the second most northerly county in Sweden.

Women were recruited through advertisements in local media and with the assistance of midwives within antenatal care. Pregnant women were recruited during the first or second trimester (at 8-16 weeks of gestation) and studied during the third trimester (at 28-32 weeks of gestation). All pregnant women had successful deliveries, one of which yielded twins. Information concerning the delivery was collected with the mothers' consent from the hospital-based register on antenatal care and delivery care. All participants provided written informed consent. The research conformed to the Helsinki Declaration and all local laws and the study protocol was approved by the Regional Ethical Review Board in Umeå.

### Assessment of body composition

Height was measured to the nearest 0.5 cm using a calibrated wall-mounted stadiometer and weight to the nearest 0.1 kg using a calibrated digital scale. Body mass index (BMI) was calculated as weight (kg) divided by height squared (m^2^) (kg/m^2^). Body composition (fat and fat-free mass) was estimated from the doubly-labeled water (DLW) measurement using the isotope dilution method [14]. Total body water was calculated as the average of the linearly regressed isotope dilution spaces at time 0, correcting by 1.01 and 1.04, respectively, to account for the exchange of isotopes with non-aqueous components within the body. Fat-free mass was then calculated by dividing by the hydration factor of 0.722 and 0.747 for non-pregnant and pregnant women, respectively, with the difference between body weight and lean tissue equating to the fat mass [15].

### Energy expenditure

Resting energy expenditure (REE) was estimated using expired gas indirect calorimetry during 30 minutes of reclined rest using a ventilated hood system (Deltatrac II, Datex-Ohmeda, Inc., WI, USA). Total energy expenditure (TEE) was estimated during ten days of free-living using DLW. For this measurement, participants received a body weight dependent oral dose of stable isotope ((0.07 g ^2^H_2_O and 0.174 g H_2_^18^O)/kg body weight). A pre-dose urine sample was obtained at the Clinical Research Center after which a urine sample was collected every day during the following ten days; participants noted the time of each sample collection in a log. Samples were stored at 4-8°C until returned to the Clinical Research Center, where they were stored at -20°C pending analysis. Urine samples were analyzed at the MRC Human Nutrition Research, University of Cambridge, Cambridge, UK, using methods described in detail elsewhere [16]. Briefly, measurements of H/H ratios were made by dual inlet isotope-ratio mass spectroscopy (IRMS) (Sira 10, VG Isogas, Middlewich, UK). Samples (0.4 ml) were aliqoted into nominally 2.5 mL vials, and a platinum catalyst added. The vials were flush filled with hydrogen gas and equilibrated at 22°C for 6 hours before analysis. All measurements were calculated relative to Vienna standard mean ocean water (V-SMOW) using international standards. Measurements of ^18^O/^16^O ratios were made using an AP2003 continuous flow IRMS (Analytical Precision Ltd, Northwich, Cheshire, UK). Samples (0.5 ml) were flush filled with 5% CO_2 _in nitrogen and then equilibrated overnight. Sample enrichments were expressed relative to V-SMOW, using laboratory standards. TEE was calculated as described by Coward et al. [17] from slopes and intercepts of the isotope disappearance curves based on urine samples collected at days 1-3 and 8-10. Respiratory quotient (RQ) was assumed to be 0.85. PAEE was calculated as (TEE × 0.9) - REE assuming 10% diet-induced thermogenesis of TEE.

### Physical activity subcomponents and sedentary time

The physical activity subcomponents, time spent in moderate-to-vigorous physical activity, total volume of activity (accelerometry counts per day) and sedentary time, were measured using heart rate and accelerometry data from an Actiheart monitor, a combined heart rate and movement sensor (CNT Ltd, Papworth, UK). The Actiheart was positioned using electrocardiogram electrodes on the left side of the chest at the level of the heart (I2-I3 level). The monitor was worn day and night during the same 10-day period as the DLW measurement was performed. Participants were asked to remove the Actiheart during periods of water immersion such as swimming or bathing. Only days with > 90% valid heart rate data were used and a minimum of four days of valid data was required in order for the analyses to be performed. Total accelerometry counts adjusted for wear time were used as an index of total volume of physical activity. Time spent in moderate-to-vigorous intensity activity was calculated as the percent of wear time with a valid heart rate greater than 174% of resting heart rate [18]. Sedentary time was estimated using epochs with valid heart rate data and zero accelerometry counts per minute and expressed as a percent of wear time.

### Blood analyses

The participants attended the Clinical Research Center on the morning following an overnight fast (≥10 h). Capillary plasma glucose was measured on a Hemocue Glucose 201 Analyzer (Hemocue, Inc., CA, USA) to ensure that women who had very low or high (i.e. consistent with a diagnosis of diabetes) blood glucose concentrations were not exposed to the glucose challenge. One woman was excluded on the basis of having high blood glucose levels. A standard 75 g oral glucose tolerance test (OGTT) [19] was performed with venous blood samples drawn prior to the challenge and at 30, 60 and 120 minutes after the glucose load. Serum insulin concentrations were determined by microparticle enzyme immunoassay (Abbott Imx, Abbott Laboratories, Abbott Park, IL) and glucose levels were analyzed using on VITROS 5,1 FS Chemistry System (Ortho-Clinical Diagnostics, Raritan, NJ) at the clinical biochemistry laboratory at Umeå University Hospital. Additional venous blood was drawn into EDTA-plasma and serum tubes and stored at -80°C for later analyses.

### Estimates of insulin sensitivity and β-cell response

We calculated the insulin sensitivity index (ISI_OGTT_) from the OGTT using the composite model of Matsuda [10000/√(Glu_0 _* Ins_0_) * (Glu_mean _* Ins_mean_)] which has been shown to be strongly correlated with insulin sensitivity measured by the hyperinsulinemic-euglycemic clamp during pregnancy [20]. The Stumvoll first-phase insulin response, calculated as [1,194 + (4.724*Ins_0_) - (117.0*Glu_60_) + (1.414*Ins_60_)] has been used previously in pregnant women [21]. β-cell function was estimated using oral disposition index (DI_O_) calculated as [(ΔIns _0-30_/ΔGlu _0-30_) × 1/fasting insulin] [22]. Insulin area under the curve (IAUC) and glucose area under the curve (GAUC) were calculated using the trapezoidal method.

### Self-reported pain

To compare pain in pregnant and non-pregnant women, participants were asked about general pain using a questionnaire. Participants were asked to rate pain during the past four weeks as well as the extent to which pain interfered with daily activities. Participants could report none, very little, light, moderate, or severe pain during the past four weeks (i.e. regarding pregnant women: during gestational weeks 24-28). Participants could also report that the pain interfered not at all, a little, moderately, or very much with daily activity (including physical activity).

### Statistical analyses

Participant characteristics are given as means and standard deviations, or as medians and interquartile range for non-normally distributed data. Comparisons between pregnant and non-pregnant women were made with t-tests or in the case of non-normally distributed data, with Wilcoxon-Mann-Whitney tests. Differences in subjective assessments of pain and the impact of pain on daily activity were assessed with Cochran-Armitage Trend tests. Insulin sensitivity or β-cell response estimates and physical activity subcomponents were adjusted for age prior to comparisons. Partial r^2 ^values were calculated from linear regression. Logarithmic transformation was used to approximate a normal distribution when necessary. Correlations were assessed with Spearman correlations. Differences in the correlations between the physical activity subcomponents and the insulin sensitivity and secretion variables were compared with tests of the equality of correlation coefficients for independent samples [23]. All statistical analyses were performed using SAS (SAS v9.2, Cary, NC).

## Results

Participant characteristics are shown in Table 1. Thirty-five pregnant women and 73 non-pregnant women entered the study. Energy expenditure data from DLW were unavailable for three women (all non-pregnant). Two pregnant women dropped out during the course of the study and valid Actiheart data could not be retrieved from the monitors for one pregnant woman and one non-pregnant woman, thus 32 pregnant and 69 non-pregnant women had complete datasets. The pregnant women were significantly older than the non-pregnant women. REE was significantly higher in pregnant women. The two groups did not differ significantly in height, weight, BMI, or in other estimates of body composition (percent body fat, fat mass, or fat-free mass).

**Table 1:** Demographic and anthropometric characteristics

	**Pregnant**	**Non-pregnant**	**p-value^a^**
	
N	32	69	
Age (years)	30.4 (2.9)	28.6 (4.4)	0.013
Height (m)	1.67 (0.06)	1.67 (0.07)	0.750
Weight (kg)	76.1 (14.1)	76.7 (19.2)	0.809
BMI (kg/m^2^)	27.4 (5.0)	27.4 (6.5)	0.987
Fat mass (kg)	26.4 (11.0)	29.2 (14.7)	0.281
Fat-Free mass (kg)	49.7 (4.7)	47.5 (6.1)	0.067
Body fat (%)	33.6 (6.8)	36.0 (9.3)	0.190
TEE (kJ/day)	11185 (1176)	11068 (1582)	0.706
REE (kJ/day)	6685 (749)	6229 (825)	0.007

Statistics are means (standard deviations). ^a^p-value for t-test comparing differences between pregnant and non-pregnant women. Measurements in the pregnant women were at gestational weeks 28-32.

### Self-reported pain

Pregnant women did not differ from non-pregnant women in self-reported severity of pain during the previous four weeks, although the test for difference approached statistical significance (p = 0.051 for trend). Pregnant women reported that pain did interfere with daily activity to a greater extent than non-pregnant women (p < 0.0001 for trend).

### Physical activity subcomponents

Table 2 shows the descriptive statistics and comparisons of physical activity subcomponents between pregnant and non-pregnant women. Sedentary time was significantly higher in pregnant than in non-pregnant women. PAEE, time spent in moderate-to-vigorous intensity activity and total activity were significantly lower in pregnant compared with non-pregnant women.

**Table 2:** Physical activity subcomponents

	**Pregnant**	**Non-pregnant**	**p-value^a^**
	
*Physical activity subcomponents*			
Moderate-to-vigorous time (% weartime)	5.4 (15.3)	18.6 (14.0)	< 0.0001
Accelerometry (thousand counts per day)	30.7 (12.0)	54.6 (36.4)	< 0.0001
Sedentary time (% weartime)	55.5 (11.9)	49.2 (10.7)	< 0.0001
PAEE (kcal/day)	812 (235.4)	924 (296)	0.045
*Insulin and glucose variables*			
Fasting Glucose (mmol/L)	4.0 (0.5)	4.4 (0.5)	< 0.0001
30 Minute Glucose (mmol/L)	6.1 (1.5)	6.4 (1.5)	0.2422
60 Minute Glucose (mmol/L)	6.6 (1.8)	5.8 (2.4)	0.2859
120 Minute Glucose (mmol/L)	4.8 (1.6)	5.2 (1.7)	0.392
Mean Glucose (mmol/L)	5.4 (1.1)	5.4 (1.3)	0.3049
GAUC (mmol/L)	186.0 (148.5)	141.0 (180.0)	0.0461
Fasting Insulin (pmol/L)	50.0 (42.4)	34.0 (34.0)	0.0021
30 Minute Insulin (pmol/L)	444.5 (243.1)	305.6 (222.2)	0.0069
60 Minute Insulin (pmol/L)	562.5 (382.0)	319.5 (229.2)	0.0004
120 Minute Insulin (pmol/L)	46.0 (36.0)	27.0 (24.0)	0.012
Mean Insulin (pmol/L)	336.1 (202.8)	201.4 (143.8)	0.0026
IAUC (pmol/L)	6049.5 (3298.5)	3710.3 (2974.5)	0.0031

Descriptive statistics are medians (interquartile range); PAEE, physical activity energy expenditure; GAUC, incremental glucose area under the curve; IAUC, incremental insulin area under the curve. ^a^p-value for Wilcoxon-Mann-Whitney test comparing differences between pregnant and non-pregnant women.

### Insulin and glucose measures

Table 2 shows the insulin and glucose measures used to calculate the estimates of insulin sensitivity and β-cell response. At fasting, 30, 60, and 120 minutes post glucose load, insulin levels were significantly higher in pregnant women than in non-pregnant women. Fasting glucose was significantly lower in pregnant women, but at 30, 60, and 120 minutes post-load glucose was not significantly different between pregnant and non-pregnant women. Pregnant women had significantly higher incremental insulin and mean insulin levels, and a larger glucose area under the curve than non-pregnant women.

### Insulin sensitivity and β-cell response estimates

Estimated first-phase insulin response was significantly higher in pregnant women than in non-pregnant women (p = 0.035). Insulin sensitivity index (ISI_OGTT_) was significantly lower in pregnant women (p = 0.016). There were no significant differences in β-cell function in relation to insulin sensitivity during an oral glucose challenge (DI_O) _(p = 0.72).

### Physical activity subcomponents and measures of insulin sensitivity and secretion

The correlations and partial r^2 ^values between total activity, PAEE, moderate-to-vigorous intensity time, sedentary time, and the measures of β-cell response and insulin sensitivity are shown in Table 3. Only total activity was significantly associated with any of the estimates of insulin sensitivity or β-cell response. Total activity was significantly positively correlated with ISI_OGTT _in non-pregnant women and was significantly negatively correlated with first-phase insulin response in both pregnant and non-pregnant women. In tests of equality of correlations comparing pregnant and non-pregnant women, the correlations did not differ significantly for any comparison between physical activity subcomponents and measures of insulin sensitivity or secretion.

**Table 3:** Relationships between physical activity subcomponents and measures of insulin sensitivity and β-cell response

	Moderate-to-Vigorous Time (% of weartime)				Total activity (counts/day)				Sedentary Time (% of weartime)				PAEE (kcal/day)			
	
	Pregnant		Non-pregnant		Pregnant		Non-pregnant		Pregnant		Non-pregnant		Pregnant		Non-pregnant	
	
	Partial r^2^	r (95% CI)	Partial r^2^	r (95% CI)	Partial r^2^	r (95% CI)	Partial r^2^	r (95% CI)	Partial r^2^	r (95% CI)	Partial r^2^	r (95% CI)	Partial r^2^	r (95% CI)	Partial r^2^	r (95% CI)
ISI_OGTT_	0.004	0.10	0.034	0.25	0.048	0.28	0.221	**0.46**	0.0003	-0.06	0.004	0.04	0.02	0.12	0.0002	-0.01
		(-0.16 to 0.34)		(-0.11 to 0.56)		(-0.07 to 0.57)		**(0.24 to 0.64)**		(-0.40 to 0.29)		(-0.21 to 0.29)		(-0.23 to 0.44)		(-0.24 to 0.24)

First phase insulin response	0.0003	-0.07	0.044	-0.16	0.108	**-0.47**	0.106	**-0.36**	0.009	0.17	0.004	-0.13	0.037	-0.22	0.011	-0.16
		(-0.41 to 0.29)		(-0.40 to 0.10)		**(-0.70 to -0.15)**		**(-0.56 to -0.12)**		(-0.18 to 0.49)		(-0.38 to 0.11)		(-0.52 to 0.12)		(-0.39 to 0.07)

DI_o_	0.005	0.03	0.001	0.02	0.021	0.24	0.007	0.10	0.002	0.25	0.0005	0.02	0.004	-0.10	0.0005	0.04
		(-0.33 to 0.38)		(-0.23 to 0.28)		(-0.12 to 0.54)		(-0.16 to 0.34)		(-0.10 to 0.55)		(-0.23 to 0.27)		(-0.42 to 0.25)		(-0.20 to 0.28)

R-values are Spearman correlation coefficients. All correlations partialled for age, PAEE additionally partialled for weight. Partial r^2 ^are from multiple linear regressions including age, and for PAEE, age and weight as covariates. PAEE, physical activity energy expenditure; ISI_OGTT_, Matsuda composite insulin sensitivity index; DI_O_, oral disposition index. **Bold text **indicates a statistically significant (p < 0.05) correlation.

## Discussion

This is the first study to our knowledge to use gold-standard methods to assess relationships between physical activity and glucose and insulin dynamics in pregnancy. Our main findings are that although the levels of physical activity differ between pregnant and non-pregnant women, with the latter being most active, the impact of physical activity on estimated insulin sensitivity and β-cell response did not differ between groups. These findings are important, because they highlight specific dimensions of physical activity that can be intervened upon and that are likely to promote improvements in a woman's insulin and glucose dynamics during pregnancy, thus aiding in the prevention of GDM.

Consistent with studies published elsewhere, we found that insulin sensitivity is lower during pregnancy and β-cell response is higher [24]. We also found that, compared with non-pregnant women, pregnant women were less active as measured by most of the subcomponents of physical activity. Specifically, pregnant women engaged in less total activity, had lower PAEE, spent less time undertaking moderate-to-vigorous intensity activity, and spent more time sedentary. A key finding of our study is that despite the differences in physical activity, insulin sensitivity, and β-cell response, the associations between the physical activity subcomponents and insulin sensitivity and β-cell response do not appear to differ significantly in pregnant versus non-pregnant women. Therefore, it is likely that maintaining total physical activity through behaviors such as daily walking and limiting sedentary time has similar beneficial effects on glucose metabolism in pregnant women as in non-pregnant women.

An insufficient increase in β-cell response to compensate for the decline in insulin sensitivity, which is typical in pregnancy, is a risk factor for GDM [21]. In the present study of women with normal glucose tolerance, pregnant women had a higher β-cell response and a lower level of insulin sensitivity. Accelerometry counts per day, a measure of total physical activity, was positively related to insulin sensitivity and negatively related to β-cell response. These relationships were not significantly different in pregnant versus non-pregnant women. Insulin response relative to insulin sensitivity during an OGTT (DI_O_) was not significantly different between pregnant and non-pregnant women and no relations to physical activity subcomponents were observed. The lack of a statistically significant relationship between DI_O _and the physical activity measures suggests that in healthy pregnant women, differences in physical activity do not result in differences in glucose tolerance, although it is important to note that our study was probably underpowered to detect small effects; as such, we cannot rule out the possibility that such relationships exist.

Our results suggest that total activity is the most strongly associated component of physical activity with measures of insulin sensitivity and β-cell response in both pregnant and non-pregnant women. Other physical activity subcomponents such as sedentary time, and PAEE did not appear to be strongly associated with measures of insulin sensitivity or response. This is consistent with studies that included only non-pregnant adults [4]. This suggests that the total volume of activity both preconception as well as during pregnancy might be the most important physical activity subcomponent to emphasize for maintaining normal glucose tolerance in pregnancy. Longitudinal studies of the subcomponents of physical activity in relation to changes in insulin sensitivity and β-cell response during pregnancy are needed. Qualitatively, the pregnant women in our study more frequently reported pain severe enough to disturb normal daily activity, which may well help explain why pregnant women were often less active than non-pregnant women. Thus, interventions that also focus on coping with pregnancy-related pain may further enhance activity levels, and hence indirectly benefit the health of the woman.

In a separate analysis testing the validity of a wrist-worn accelerometer (GENEA, Unilever Discover, UK) in this cohort, REE, PAEE, and accelerations (as measured by that monitor) were not significantly different in pregnant versus non-pregnant women (Van Hees et al, unpublished). This differed from our finding that PAEE and REE were significantly different in pregnant and non-pregnant women (p = 0.045 and p = 0.007, respectively), which is due to slight differences in the study sample. The Van Hees et al analysis included six fewer participants (4 non-pregnant and 2 pregnant) due to an absence of critical measurements that were the central feature of that study. Nevertheless, when these participants are excluded from our analysis the differences in moderate-to-vigorous time, sedentary time, and total activity remained statistically significant (data not shown).

This study is the first to our knowledge to incorporate very detailed objective measures of physical activity during pregnancy and compare these with well-characterized glucose regulation phenotypes. Our study also has limitations; first, the cross-sectional nature of the comparisons between physical activity subcomponents and estimates of insulin sensitivity and β-cell response makes it impossible to determine the direction of causality in the relationships we report. A second limitation is that because we used a range of very detailed measurement methods that are infeasible for use in large sample collections, the study sample is relatively small, which means that the detection of small effects in this study, whilst not impossible, may be unlikely.

## Conclusion

In summary, we have conducted an intensively phenotyped study of pregnant and non-pregnant women to investigate the relationships between physical activity subcomponents and estimates of insulin sensitivity and secretion. A key finding of this study is that, as previously established in non-pregnant women, total physical activity is positively associated with estimated insulin sensitivity during pregnancy. This in turn suggests that increased habitual physical activity at any intensity (rather than increased moderate-to-vigorous time) may be a means to help control glucose homeostasis during pregnancy. Appropriately designed intervention studies are required to determine whether these relationships are causal.

## Competing interests

The authors declare that they have no competing interests.

## Authors' contributions

JP analyzed the data and wrote the first draft of the manuscript with AG and PWF designed the study and FR and PWF researched data. SEK provided important guidance on the plan for data analyses. All authors reviewed/edited and provided critical feedback on the manuscript.

## Pre-publication history

The pre-publication history for this paper can be accessed here:

http://www.biomedcentral.com/1471-2393/11/44/prepub
